# Hydrogen-Induced Topotactic Phase Transformations of Cobaltite Thin Films

**DOI:** 10.1021/acs.jpcc.4c04098

**Published:** 2024-09-30

**Authors:** Mingzhen Feng, Junjie Li, Shenli Zhang, Alexandre Pofelski, Ralph El Hage, Christoph Klewe, Alpha T. N’diaye, Padraic Shafer, Yimei Zhu, Giulia Galli, Ivan K. Schuller, Yayoi Takamura

**Affiliations:** † Department of Materials Science and Engineering, University of California Davis, Davis, California 95616, United States; ‡ Department of Physics, University of California San Diego, La Jolla, California 92093, United States; § Materials Science and Engineering Program, University of California San Diego, La Jolla, California 92093, United States; ∥ Materials Science Division, Lawrence Livermore National Laboratory, Livermore, California 94550, United States; ⊥ Condensed Matter Physics and Materials Science Department, Brookhaven National Laboratory, Upton, New York 11973, United States; # Advanced Light Source, Lawrence Berkeley National Laboratory, Berkeley, California 94720, United States; ∇ Pritzker School of Molecular Engineering, University of Chicago, Chicago, Illinois 60637, United States

## Abstract

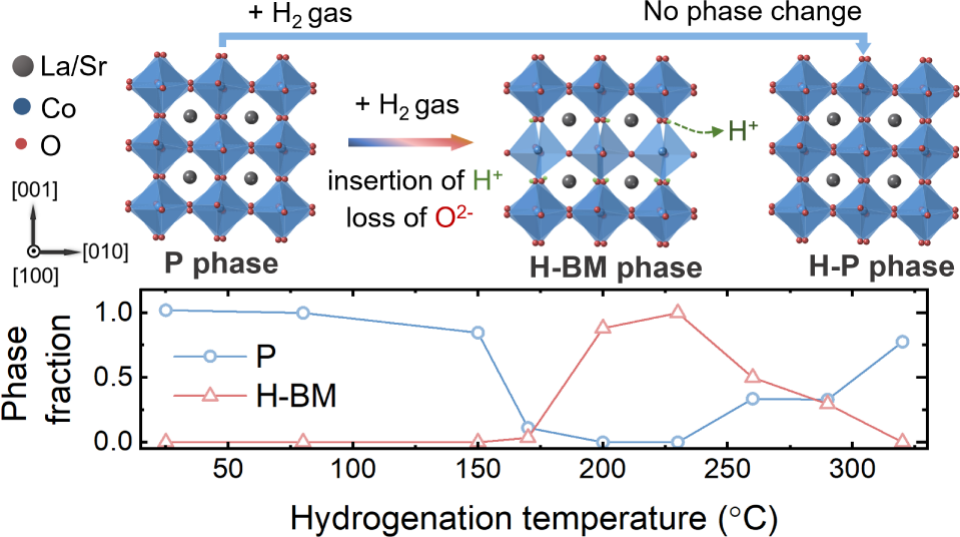

Manipulating physical properties through ion migration in complex oxide thin films is an emerging research direction to achieve tunable materials for advanced applications. While the reduction of complex oxides has been widely reported, few reports exist on the modulation of physical properties through a direct hydrogenation process. Here, we report an unusual mechanism for hydrogen-induced topotactic phase transitions in perovskite La_0.7_Sr_0.3_CoO_3_ thin films. Hydrogenation is performed upon annealing in a pure hydrogen gas environment, offering a direct understanding of the role that hydrogen plays at the atomic scale in these transitions. Topotactic phase transformations from the perovskite (P) to hydrogenated-brownmillerite (H-BM) phase can be induced at temperatures as low as 220 °C, while at higher hydrogenation temperatures (320–400 °C), the progression toward more reduced phases is hindered. Density functional theory calculations suggest that hydroxyl bonds are formed with the introduction of hydrogen ions, which lower the formation energy of oxygen vacancies of the neighboring oxygen, enabling the transition from the P to H-BM phase at low temperatures. Furthermore, the impact on the magnetic and electronic properties of the hydrogenation temperature is investigated. Our research provides a potential pathway for utilizing hydrogen as a basis for low-temperature modulation of complex oxide thin films, with potential applications in neuromorphic computing.

## Introduction

The development of neuromorphic computing has inspired a vigorous exploration of innovative materials and methodologies capable of leveraging topotactic phase transformations to tailor functionalities for information processing and memory storage.^1−7^ Topotactic phase transformations are characterized by the modulation of crystal structures involving the loss or gain of ions, providing potential pathways for tuning electrical and magnetic properties.^8−10^ Specifically, complex oxide thin films offer diverse functionalities including metal–insulator transitions (MIT), ferromagnetism, and superconductivity that stem from complex interplays between the spin, charge, orbital, and lattice degrees of freedom.^11−14^ For example, strontium iridate, an oxide that offers different topotactic phases, including SrIrO_3_, Sr_2_IrO_4_, and SrIr_2_O_6_, has been widely explored for spintronic and electrocatalytic applications.^15,16^ Another prominent example, the infinite-layer nickelate (Nd_0.8_Sr_0.2_NiO_2_), synthesized by reducing the perovskite precursor phase, has recently been found to exhibit superconductivity.^14^ For selected oxide systems, the thin film geometry enables the initiation of topotactic phase transformations by a variety of approaches which are not achievable with bulk materials, such as thermal treatment in an oxygen-rich or deficient environment,^6,7,17−19^ depositing metal getter layers which leach oxygen from the film,^20−22^ or varying the initial growth condition of a thin film.^23,24^ These methods offer limited reducing capabilities. For instance, thermal treatment usually requires high temperatures (400–900 °C) to break strong metal–oxygen (M–O) bonds, thereby limiting the material compatibility in thermal-sensitive applications such as Si-based electronics^25^ or polymers.^26^ Recently, hydrogenation has been shown to offer an alternative route for tuning complex oxide thin film properties in which the interactions with hydrogen atoms/ions act as the driving force for topotactic phase transformations.^27−30^ However, a systematic study of the direct hydrogenation effect in the complex oxide phase and property change remain limited.

While the fundamental mechanisms of the hydrogenation process for complex oxides are still in debate, two primary mechanisms regarding the role of hydrogen atoms/ions have been proposed. Mazza et al. studied the hydrogenation of La_0.7_Sr_0.3_MnO_3_ (LSMO) thin films with Pt nanoparticles (NPs) on the surface as catalysts upon annealing in a gas mixture of 5% H_2_ in N_2_ at atmospheric pressure.^29,30^ Hydrogen atoms absorbed on thin film surfaces facilitate the formation of oxygen vacancies within the bulk oxide, which enables the perovskite (P) to brownmillerite (BM) phase transformation. In their work, Pt nanoparticles were required as catalysts which enable the phase transformation.^29,30^ In the second mechanism, hydrogen ions interact with the lattice ions of the complex oxide, forming direct bonds with transition metal (M) ions (i.e., substituting for O ions and forming M–H bonds)^31^ or with lattice oxygen ions to form O–H bonds.^5^ In addition, electric fields have been used to aid the insertion of O^2–^ or H^+^ ions in the reversible phase transformation of BM (SrCoO_2.5_) to P (SrCoO_3_) or H-BM (HSrCoO_2.5_).^2^ It is worth noting that hydrogen atoms can also occupy interstitial sites in perovskite thin films, for example, SmNiO_3_, leading to a significant change of their electrical properties, even without any phase transformations.^32,33^ Therefore, understanding the fundamental mechanism of hydrogen-modified complex oxides can be a challenging task.

Among complex oxides, cobaltite thin films are attractive due to their diverse array of perovskite-related phases, each characterized by unique magnetic and electrical properties, rendering them promising materials for neuromorphic computing applications.^2,7,17,34−36^ For example, the manipulation of distinct topotactic phases of SrCoO_*x*_ (SCO) thin films results in a tailored evolution of its physical properties from the equilibrium antiferromagnetic (AFM)/insulating BM phase (SrCoO_2.5_) to the metastable ferromagnetic (FM)/conducting P phase (SrCoO_3_).^17,37^ In a previous work,^7^ a series of topotactic transformations were initiated in La_0.7_Sr_0.3_CoO_3_ (LSCO) thin films that grow on (LaAlO_3_)_0.3_(Sr_2_TaAlO_6_)_0.7_ (LSAT) substrates with controlled reducing conditions through thermal vacuum annealing. BM and Ruddlesden–Popper (RP) phases were obtained in high-temperature (≥400 °C) and ultrahigh vacuum (*p*o_2_ = 10^–12^ – 10^–24^ atm) conditions.

In this work, we explore direct hydrogenation that utilizes a pressurized pure hydrogen gas environment (0.3 MPa) as an alternative approach to induce topotactic transformations in epitaxial LSCO thin films under moderate temperature conditions (<400 °C). The simplicity of this hydrogen-oxide system provides a straightforward platform where density functional theory (DFT) calculations and experiments can be directly compared. It is found that a topotactic phase transformation from the P to hydrogenated-BM (H-BM) phase can be induced by direct hydrogenation at temperatures as low as 220 °C (compared to 400 °C under vacuum annealing conditions), due to the reduction of the oxygen vacancy formation energy with the presence of hydrogen in the lattice. However, the transition to the RP or other reduced phases was not observed at higher temperatures, and instead, the thin film remained in the P phase. DFT calculations suggest that this is due to a higher formation energy of oxygen vacancies in the presence of hydroxyl bonds compared to neighboring oxygen ion sites that are not bonded to H ions. The structural, magnetic, and electronic properties of the LSCO thin films after hydrogenation at different temperatures were experimentally investigated. Our research contributes to the fundamental understanding of hydrogenation processes in complex oxide thin films, providing a potential pathway for low-temperature modulation of oxide materials among topotactic phases.

## Experimental Methods

### Thin Film Deposition

The LSCO thin films with around 20 nm thickness were grown on (001)-oriented single-side polished LSAT substrates by pulsed laser deposition from a stoichiometric La_0.7_Sr_0.3_CoO_3_ target. For consistency and to minimize potential variations in film quality, four 5 × 5 mm^2^ substrates were mounted at the central region of the heater plate during a single growth. The chamber was pumped to a base pressure of 4 × 10^–4^ Pa and then subsequently filled with flowing O_2_ gas to establish a constant 40 Pa pressure. During the deposition, the substrate temperature was held at 700 °C, and a KrF excimer laser (λ = 248 nm), with 0.8 J/cm^2^ laser energy and a 1 Hz laser repetition rate, was used. The thin film samples were cooled to room temperature in a 4 × 10^4^ Pa oxygen environment at a cooling rate of 10 °C/min to ensure proper oxygen stoichiometry, which is essential in the study of topotactic phase transformations.

### Hydrogenation

Hydrogenation was performed in a homemade rapid thermal hydrogenation system previously reported.^38^ The hydrogenation setup consists of a thickened quartz tube connected to a vacuum chamber capable of achieving hydrogen pressures from 0.13 to 5 × 10^5^ Pa and temperatures up to 1000 °C. Before flowing hydrogen gas, the quartz tube was preheated to the hydrogenation temperature and maintained at ∼1.33 × 10^–4^ Pa base pressure for at least 30 min to remove any potential gas impurities. A high vacuum transfer arm was used to insert the LSCO film into the quartz tube. Once the film was loaded and the arm was retracted, ultrahigh purity hydrogen gas (>99.999%) was introduced into the tube at a rate of ∼0.06 MPa/s using a needle valve and held at a constant pressure of 0.3 MPa. After 1 h of hydrogenation (the same duration as in the previous study on vacuum annealing),^7^ the tube was depressurized at a rate of ∼0.04 MPa/s and pumped down to high vacuum. Subsequentially, the film was extracted using the same transfer arm, which was capable of cooling the sample at an approximate rate of ∼10 °C/s. The sample was settled for at least 5 min in high vacuum before being exposed to air.

### Structural Characterization

High-resolution X-ray diffraction (XRD) measurements were conducted at room temperature using a Rigaku SmartLab diffractometer with Cu Kα_1_ radiation (λ = 0.154 nm).

### STEM and EELS

A thin layer of Au was first deposited on top of the hydrogenated-LSCO thin film sample to avoid charging during the sample preparation. The sample was then prepared using the in situ lift-out method in a Helios G5 DualBeam instrument operating at 30 keV. A final cleanup at 5 keV was performed to remove potential amorphous material on both sides of the lamellas. The sample was imaged at room temperature in a double aberration corrected JEOL ARM 200F cold FEG microscope operating at 200 keV. The high-angle annular dark field scanning transmission electron microscopy (HAADF-STEM) imaging was performed with a semiconvergence angle of 21 mrad and a current of approximately 60 pA resulting in a probe size of around 80 pm. The SmartAlign plug-in for the Gatan Microscopy Suite Software v3.5 was used to remove the scanning distortions in the multiframe data set.^39^ The electron energy loss spectroscopy (EELS) analysis was performed using the GIF Continuum spectrometer and the K3 direct detection camera in Dual EELS counting mode using a dwell time of 50 ms (5 and 45 ms for the low loss and core loss part, respectively). The zero-loss peak was used to align the spectral mappings along the energy axis. The core loss EELS spectra were denoised using the principal component analysis method (PCA),^40^ keeping the first 15 components.

### Valence State and Bonding Configurations

Soft X-ray absorption (XA) and X-ray magnetic circular dichroism (XMCD) spectra were acquired at the Co *L*-edge at 80 K using Beamline 4.0.2 of the Advanced Light Source (ALS) in total electron yield (TEY) and luminescence yield (LY) detection modes. TEY detection probes the sample surface (top 5–10 nm) due to the limitation of the escape length of secondary electrons,^41^ while LY detection provides information on the entire film thickness.^42^ For XMCD measurements, the films were field-cooled to 80 K in 0.3 T magnetic field to ensure that all of the magnetic moments are aligned along the field direction. During the measurements, a 0.3 T magnetic field was applied parallel to the incident X-ray beam, which was 60° from the surface normal. XMCD spectra were calculated as the difference between two jointly normalized soft XA spectra collected with right (*I*_RCP_) and left (*I*_LCP_) circularly polarized X-rays.^43^ Oxygen *K*-edge XA spectra were acquired at room temperature using Beamline 6.3.1 of the ALS in TEY mode with no magnetic field applied.

### DFT Calculations

First-principles calculations were performed using the Quantum Espresso code (v7.2),^44,45^ which solves the Kohn–Sham equations of DFT using plane waves and pseudopotentials. More specifically, we used DFT + *U* (*U* = 3 eV as justified in previous calculations),^4^ with the Perdew–Burke–Ernzerhof (PBE) generalized gradient approximation^46^ for the exchange-correlation functional and the projector augmented wave pseudopotentials from the PSlibrary^47^ (v1.0.0 for La, Sr, H and O and v0.3.1 for Co). A 40-atom orthorhombic cell with a La_0.625_Sr_0.375_CoO_3_ stoichiometry was used to study the formation energies of hydrogen and oxygen vacancies. A 1224 eV plane-wave cutoff and a Monkhorst–Pack *k*-point grid^48^ with the resolution between 0.02 and 0.03 Å^–1^ were used. The convergence thresholds for energy, force, and pressure for structural optimization were set to 8.5 × 10^–5^ eV per formula unit (f.u.), 2.5 × 10^–4^ eV Å^–1^, and 0.5 Kbar, respectively.

### Functional Property Characterization

Bulk magnetic properties were determined by using a Quantum Design VersaLab or DynaCool system equipped with a vibrating-sample magnetometer. A magnetic field of 0.08 T was applied along the in-plane [100] substrate direction as the sample was cooled to 50 K. Magnetization vs temperature (*M*(*T*)) measurements were performed upon warming from 50 to 350 K, with 0.08 T magnetic field along the same direction. Measurements of film resistivity were conducted by using a Lakeshore cryogenic probe station with four-point van der Pauw geometry. The resistivity was measured upon warming from 50 to 350 K, with a controlled heating rate of approximately 3 K/min.

## Results and Discussion

**Figure 1:**
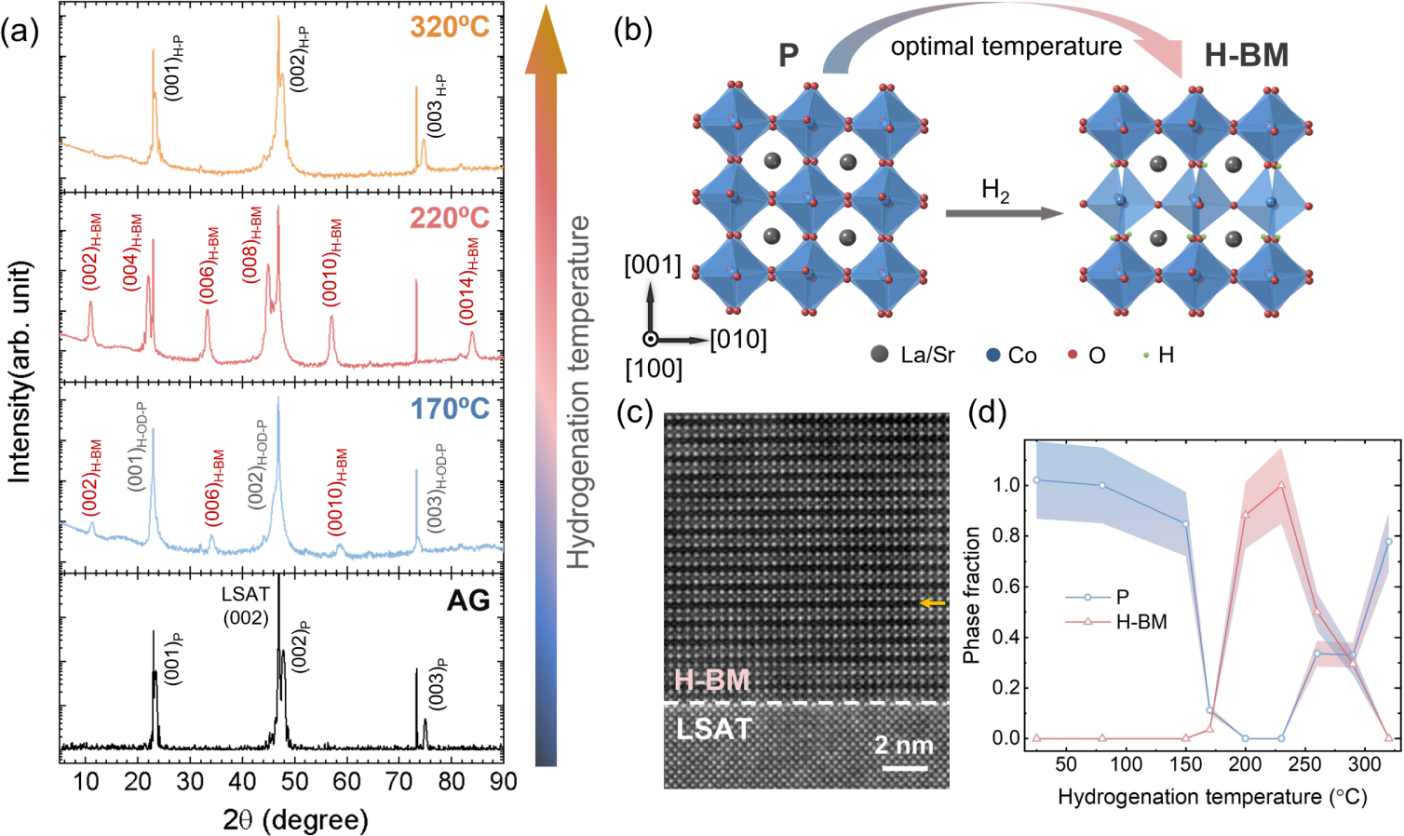
(a) XRD patterns of AG-P and hydrogenated-LSCO thin films on LSAT substrates upon annealing for 1 h. The hydrogenation temperature increases from bottom to top. (b) Crystal structure diagrams of the P and H-BM phases. La/Sr, Co, and H ions are shown in gray, blue, red, and green, respectively. The structures are oriented along the [100]-pseudocubic axes of the P phase. (c) STEM-HAADF image of the H-BM phase after annealing at 220 °C. The yellow arrow marks the horizontal dark stripe (oxygen-deficient layer). (d) Phase fraction of P and H-BM phases for hydrogenated samples as a function of the hydrogenation temperature. The shaded region denotes the range of error bars.

Figure 1a presents the ω*-*2θ XRD curves of ∼20 nm LSCO thin films grown on LSAT substrates that were exposed to 1 h hydrogenation annealing at temperature of 170 °C, 220 °C, and 320 °C. These three representative temperatures are selected from a more comprehensive series of XRD patterns spanning a wider range of hydrogenation temperatures (from 80 to 400 °C) as shown in Figure S1. A series of (00*L*) peaks can be observed, and from their periodicity and 2θ values, they can be ascribed to the P phase, hydrogenated oxygen-deficient perovskite (H-OD-P) phase, or hydrogenated-BM (H-BM) phase, where the H-index specifies a phase obtained from hydrogenation rather than vacuum annealing. In particular, the H-BM phase is characterized by a series of so-called “half-order peaks” present between the integer peaks of the perovskite LSAT substrates (i.e., 2θ values of 11.21°, 34.17°, and 58.73°) which correspond with the quadrupling of the unit cell due to the alternating octahedrally and tetrahedrally coordinated layers (see crystal structures with alternating layers shown in Figure 1b). Bulk LSCO films have a lattice parameter of 3.833 Å in pseudocubic notation, while the cubic LSAT substrate has a lattice constant of 3.867 Å.^49^ Therefore, the film has an in-plane tensile strain of 0.89%, where the strain is denoted by ε = (*a*_strained_ – *a*_bulk_)/*a*_bulk_. As shown in Figure 1a, the as-grown (AG)-P LSCO film peaks are on the high-angle side of the LSAT substrate peaks, corresponding to smaller out-of-plane lattice constants (3.80 Å) as expected. Upon hydrogenation at 170 °C, the integer film peaks shift toward lower 2θ angles. This shift corresponds to an expansion of the out-of-plane lattice constant (from 3.80 to 3.93 Å), which implies the formation of oxygen vacancies in the H-OD-P phase. In addition, weak half-order peaks appear corresponding to the coexistence of H-OD-P and H-BM phases. Coexistent OD-P and BM phases have been reported in LSCO thin films with a Gd capping layer, where the BM phase has the lateral size around 0.5–1 μm and 10–75 μm in length in the nanodiffraction maps. The spontaneous oxidation of the Gd capping layer leaches O^2–^ ions from the oxide thin film.^21^ For the LSCO film hydrogenated at 220 °C, prominent half-order peaks indicate a near-complete phase transition to the H-BM phase. The atomic scale structure of this pure H-BM phase was probed using HAADF-STEM as shown in Figure 1c. This structure is characterized by alternating bright (octahedrally coordinated layers) and dark (tetrahedrally coordinated) stripes stacked along the out-of-plane direction. The dark stripes, marked by a yellow arrow in Figure 1c, result from the coalescence of oxygen vacancies in the tetrahedrally coordinated layers. The out-of-plane lattice constant is calculated to be 16.12 Å which is slightly larger than the BM phase (La_0.7_Sr_0.3_CoO_2.5_) (16.053 Å) formed after vacuum annealing at 400 °C and oxygen partial pressure *p*o_2_ = 1 × 10^–24^ atm.^7^ Unlike annealing performed under vacuum conditions,^7^ at the 320 °C hydrogenation temperature, no half-order peaks were observed, and the integer film peaks are on the high-angle side of the substrate peak. The integer film peak positions closely align with those of the AG-P LSCO film with an out-of-plane lattice constant of 3.81 Å. Therefore, we denote this high-temperature phase as the hydrogenated-perovskite (H-P) phase distinct from the AG-P or H-OD-P phases. Figure 1d shows the phase fraction determined from fitting XRD profiles using the AG-P LSCO film as the P phase and the film hydrogenated at 220 °C as the pure H-BM phase. The shaded region denotes the error bars obtained from the fitting. The evolution of the curves shows that the formation of different perovskite-related phases can be controlled by using the hydrogenation temperature. Under these conditions, the H-BM phase only forms for hydrogenation temperatures between 170 and 290 °C, and above 290 °C, instead of forming more reduced phases, the LSCO thin film forms a H-P structure.

**Figure 2:**
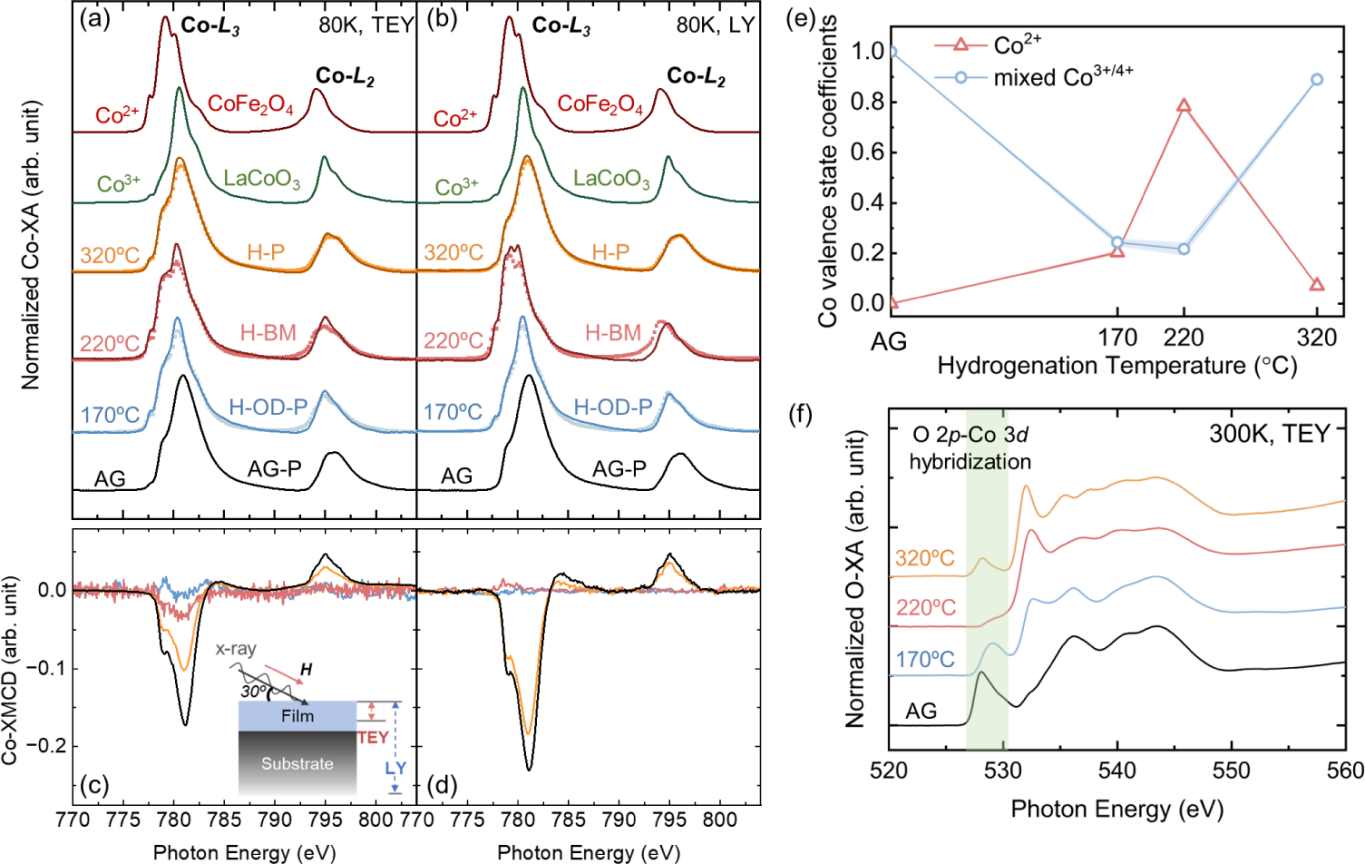
Co *L*-edge XA of AG-P and hydrogenated-LSCO thin films measured in (a) the TEY mode and (b) the LY mode. Solid curves are experimental data, and symbols are fitting results. Co *L*-edge XA reference spectra (CoFe_2_O_4_, LaCoO_3_) are plotted. XA spectra are normalized from 0 to 1 and vertically shifted for clarity. Co *L*-edge XMCD measured in (c) TEY and (d) LY mode. The inset diagram in (c) represents the measurement geometry and the probing depth of TEY and LY detection modes. (e) Co-ion valence state fitting coefficients as a function of hydrogenation temperature. (f) O *K*-edge XA spectra taken at 300 K using the TEY mode. The shaded region in green represents the energy range associated with hybridization between O 2*p* and Co 3*d* orbitals.

To gain insights into the bonding configuration of the Co, H, and O^2–^ ions, XA and XMCD spectra were acquired at the Co *L*-edge and the O *K*-edge. Figure 2a,b plots the Co *L*-edge XA spectra acquired in TEY mode (surface sensitive) and LY detection mode (offering insights throughout the entire film thickness), respectively. Both panels show a pronounced shift of the Co-*L*_*3*_ peak to lower photon energy for the H-OD-P (blue curve) and H-BM films (red curve) in comparison to the AG-P LSCO film (black curve), indicating a significant reduction in the Co-ion valence state. In addition, the small pre-edge feature (∼777.5 eV) in the H-OD-P and H-BM films and the prominent triplet structure of the Co-*L*_*3*_ main peak of the H-BM film suggest the presence of multiple Co-ion valence states. The similarities in the peak shape and photon energy with the Co^2+^ reference spectra (CoFe_2_O_4_) indicates that Co^2+^ ions are the dominant valence state in the H-BM phase. A comparison of Figure 2a,b allows us to better understand the characteristics of the film surface vs the bulk film. The Co-*L*_*3*_ peak of the H-BM film near the film surface has less peak splitting, and the peak position shifts by 0.2 eV to a higher photon energy compared to the LY spectra. This result implies that the surface region of the film is characterized by higher Co valence states than the bulk. The probing depths of TEY and LY detection modes are schematically shown in the inset of Figure 2c.

HAADF-STEM images of a H-BM film shown in Figure S2a,b further support the nonuniform phase distribution throughout the film thickness. The surface region (∼3.5 nm) exhibits similar structure to the P phase, while the bulk of the film has transformed to the BM phase. We also noticed that the film/substrate interface region (∼2 nm) maintains the P phase structure as shown in Figure S2a. EELS spectra of the Co *L*_2,3_-edges (see Figure S2c,d) present similar shifts of the peak position compared to the XA spectra as a function of distance from the film surface. However, we cannot rule out the possibility of surface oxidation, due to the sample exposure to air without a protective capping layer. At 320 °C hydrogenation temperature, the Co-XA spectrum of the H-P film closely resembles the spectrum from the AG-P LSCO sample, suggesting the comparable valence state and coordination number for the Co ions (mixed Co^3+^/Co^4+^ ions in octahedral coordination).

Figure 2c,d presents the Co-XMCD spectra measured in TEY and LY modes, respectively, derived as the difference between XA spectra acquired with right (*I*_RCP_) and left (*I*_LCP_) circularly polarized X-rays (*I* = *I*_RCP_ – *I*_LCP_). The films were field-cooled to 80 K in 0.3 T magnetic field to ensure that all of the magnetic moments are aligned along the field direction. During the measurements, the applied magnetic field (0.3 T) is oriented parallel to the direction of the X-rays and saturates the magnetic moments within the film plane. In both TEY and LY modes, the AG-P film displays strong XMCD intensity, and the H-P film shows lower magnetization, indicative of ferromagnetism from mixed Co^3+^/Co^4+^ ions. The different XMCD intensity measured in TEY and LY modes is due to the different probing depth into the film. The XMCD result corroborates the XRD findings that the H-P phase maintains the same structure and magnetic ordering as the AG-P film. It suggests that at higher temperatures, it is energetically unfavorable to form oxygen vacancies with the presence of hydrogen in the lattice. In contrast, thin films featuring the H-OD-P and H-BM phases exhibit a weak XMCD signal in TEY mode and no XMCD signal in LY mode, suggesting that the Co ions at the surface are FM, while the bulk of the film is non-FM at 80 K. Again, it agrees with the results from Figure S2a,b that the phase distribution is nonuniform vertically throughout the film thickness. With the existence of oxygen vacancies in reduced phases, the Co–O bond length and Co–O–Co bond angle can be affected, leading to the change of magnetic ordering.^50^ Previous studies on the BM phases of La_1–*x*_Sr_*x*_CoO_3−δ_ thin films, with *x* = 0.3 and 0.5, obtained by vacuum annealing, have shown a weak XMCD signal at 80 K and FM ordering (with Curie temperature *T*_c_ ≈ 115 K) by direct neutron diffraction, respectively.^7,51^ In addition, the HSrCoO_2.5_ phase obtained through ionic liquid gating, also exhibits a weak FM XMCD signal at 20 K.^2^ In contrast, the absence/weak of an XMCD signal at 80 K in the H-OD-P and H-BM phases, observed in this study, suggests that the changes in magnetic ground states during hydrogenation may result from the combined effects of oxygen loss and hydrogen insertion. However, we cannot rule out the possibility that these phases might exhibit magnetism at lower temperatures, which requires further investigation.

To quantify the fraction of Co-ion valence states, a linear fitting was applied to the Co-XA spectra of the hydrogenated films, using reference XA spectra from Co^2+^ ions (CoFe_2_O_4_), mixed Co^3+/4+^ ions (AG-P), and Co^3+^ ions (LaCoO_3_) as shown in Figure 2a,b. The solid curves of H-OD-P, H-BM, and H-P spectra are experimental data, and the symbols are fitting results. The linear fitting equation is
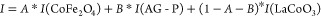
1where *I* is the XA spectra intensity in LY mode, and *A* and *B* are independent variables referred to the Co valence state coefficients as plotted in Figure 2e. Here, the analysis focused on the contributions from Co^2+^ and mixed Co^3+/4+^ ions, as shown in Figure 2e. Previous reports have shown that Co^3+^ ions are mainly confined to the interface between the film and substrate, where this region is regarded as a nonmagnetic dead layer with a thickness below 2 nm.^52,53^ An increasing trend in Co^2+^ ions and a decreasing trend in mixed Co^3+/4+^ ions are observed as the hydrogenation temperature is raised to 220 °C, suggesting that the H-BM phase predominantly comprises lower valence Co ions. Upon further temperature increase to 320 °C, the fraction of mixed Co^3+/4+^ ions approaches 1, while the proportion of Co^2+^ ions decreases significantly to nearly 0. It is important to note that the fitting qualities of the H-BM film spectra as shown in Figure 2a,b are not as optimal as other samples, and the peak shape does not align well with that of the BM-phase LSCO after vacuum annealing as previously reported.^7^ This observation leads us to propose that beyond the loss of O^2–^ ions, H ions are inserted into the film, causing the Co-XA spectral shape to differ from a conventional BM-phase XA spectra obtained from vacuum annealing.^7^ A Co-XA spectrum displaying similar triplet peaks at the Co *L*_*3*_-edge has been reported for HSrCoO_2.5_ thin films where the H^+^ ions are bonded to the apical O^2–^ ions in tetrahedral layers.^2^ Therefore, we propose that the H-BM phase is attributed to the topotactic phase transformation from a perovskite phase with not only the loss of the O^2–^ ions but also the insertion of the H^+^ ions.

Figure 2f plots the O *K*-edge XA spectra measured at 300 K using TEY detection mode, exclusively probing the oxygen environment within the film while excluding substrate signals that may be present using LY detection. The green shaded region signifies hybridization between O 2*p* and Co 3*d* orbitals within octahedral coordination,^50,54^ which is also sensitive to the existence of H^+^ ions bonded to the O^2–^ ions.^2^ The weak prepeak near 529 eV for the H-BM film corresponds to lower Co valence states, consistent with the increase in Co^2+^ ion concentration shown in Figure 2a. O *K*-edge EELS spectra of the H-BM film (see Figure S2c,d) show a similar prepeak intensity change and peak position shift between the surface P phase and bulk H-BM phase. In contrast, for the H-OD-P and H-P films, the existence of the prepeaks suggests that the films are still dominated by Co ions with higher valence states but not as much as in the AG-P film due to lower peak intensities. A discernible discrepancy in peak shapes between the AG-P and hydrogenated phases can be noticed in the photon energy range from 535 to 545 eV, suggesting different oxygen ion bonding configurations with the presence of hydrogen ions. However, the characteristic hydroxyl peak around 540 eV, reported in other studies,^2,5^ is not observed. Here, we cannot rule out the lower concentration of H^+^ ions near the surface compared to the bulk due to surface oxidation and X-ray exposure, which would require alternative techniques for quantification.

While it is difficult to observe directly the presence of H^+^ ions in the LSCO lattice, our experimental results provide several pieces of indirect evidence. XRD measurements show a small swelling of the out-of-plane lattice parameter for the hydrogenated phases, that is, from 3.80 to 3.81 Å from the P to the H-P phases and 16.053 to 16.12 Å from the BM to the H-BM phases. Analyses of the XA and EELS spectra at the Co *L*-edge and O *K*-edge suggest that the increased concentration of Co^2+^ ions in the H-OD-P and H-BM films is associated with the formation of oxygen vacancies. Additionally, the unique triplet peaks observed at the Co *L*_3_-edge of the H-BM film, together with the change in the spectral shape between 535 and 545 eV in the O *K*-edge XA spectra, suggest the insertion of H ions during hydrogenation of H-OD-P, H-BM, and H–P phases is distinct from what was observed upon vacuum annealing.

**Figure 3:**
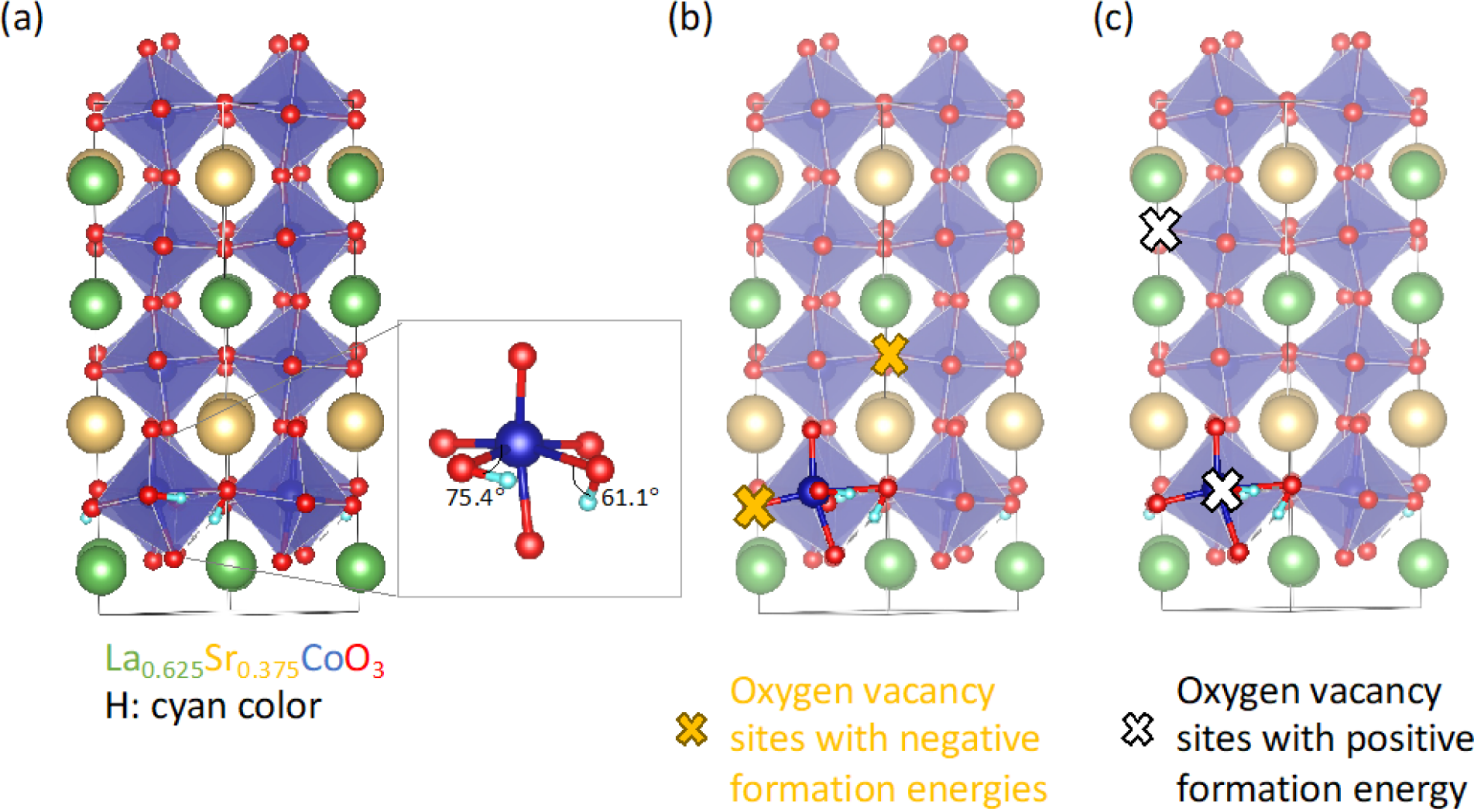
(a) Hydrogen configuration with the lowest formation energy (Δ*E*_fH_), where the H_2_ molecules decompose and form two neighboring −OH bonds. The inset figure shows details of the −OH bonding structure, with the Co–OH bond angle marked in the figure. (b) Oxygen vacancy sites with negative oxygen vacancy formation energies (Δ*E*_fO_), which are close to −OH bonds. (c) Oxygen vacancy sites with positive oxygen vacancy formation energy Δ*E*_fO_), which are bonded to hydrogen.

To understand the bonding configuration of hydrogen in the lattice and to explain the emergence of the high-temperature H-P phase, DFT calculations were performed (see the Methods section for details of the calculations and functional used). We first investigated the bonding configuration when one H_2_ molecule was introduced to a 40-atom La_0.625_Sr_0.375_CoO_3_ unit cell (the chosen La: Sr ratio is determined by the limitation imposed by the supercell cell adopted in the simulations); that is, the doping concentration is 1:8 between H_2_ and Co ions. We considered various doping positions for molecular adsorption, as well as separation of the two hydrogen atoms to form bonds, with A- (La or Sr), B- (Co), and O-sites (see Figure S3), and calculated formation energies of hydrogen doping (Δ*E*_fH_) (see Table S1). For the calculation, we used the same pressure (0.3 MPa H_2_) as used experimentally. This pressure was applied to the equation for Δ*E*_fH_ (eq S1) and the chemical potential term of H_2_ gas molecules (eq S2). We performed calculations at three different temperatures to show the effect of hydrogenation temperature and to match the experimental temperature range studied (Table S1 and related discussion). The hydrogen atoms introduced in our unit cells are charge neutral since, experimentally, hydrogenation was done through annealing in a H_2_ gas environment. In contrast, in the electric field controlling method, H^+^ ions diffuse into the material.^2^ Molecular adsorption is found to be energetically unfavorable in our calculations as the H_2_ molecule spontaneously decomposes and forms two −OH bonds during the relaxation of the atomic coordinates. Moreover, we found that two −OH bonds connecting to the same Co ion with an approximate 90° O–Co–O bond angle lead to the lowest Δ*E*_fH_ (Figure 3a), and Δ*E*_fH_ generally decreases (becomes more negative) as a function of the distance between the two −OH bonds. We also ruled out the possibility of hydrogen directly bonded to Co ions that was reported before.^31^ Specifically, we found that Co–H bonding configurations are unstable, and during structural relaxations, H detaches from the Co ions and forms an −OH bond instead. When increasing the temperature in the chemical potential term, the value of Δ*E*_fH_ further decreases (see Table S1), and thus, higher hydrogen doping concentration is expected at higher temperatures. For most of the doping configurations, we do not observe the presence of Co^2+^ ions (see Table S1), which is consistent with the valence state characterization of the H-P sample in Figure 2e.

By adopting the lowest −OH bonding configuration, we then introduced one oxygen vacancy at different lattice sites to understand the formation energy of oxygen vacancies (Δ*E*_fO_) in the presence of hydrogen. We considered several configurations, with a neutral oxygen atom removed from the −OH bonds, the nearest neighbor oxygen atom to the −OH bond, or the oxygen atoms further away from the −OH bonds. The detailed calculation procedure and results can be found in Table S2 and related discussion. The calculation results suggest that the oxygen vacancies either next to or in the neighboring CoO_6_ octahedral layers to the −OH bond have the lowest Δ*E*_fO_ between −0.2 and −0.4 eV per oxygen vacancy at 0 K (Figure 3b). The negative values here indicate the spontaneous formation of oxygen vacancies in the hydrogenation environment. However, the calculated value of Δ*E*_fO_ with the absence of hydrogen (e.g., in vacuum or in the presence of other inert gases present in annealing experiments) is about 1 eV (Figure 3c), much higher than Δ*E*_fO_ obtained in Figure 3b. These results indicate that it is less energetically favorable to form a reduced phase (such as the BM phase) if no hydrogen is present. In addition, we find that the removal of an oxygen ion in −OH bonds also has a high formation energy of about 1 eV, indicating that the formation of highly reduced phases (e.g., RP phases) in the presence of oxygen ions participating in hydroxyl bonds (when the hydrogen doping concentration is high) is energetically unfavorable. The latter case might occur at higher hydrogenation temperature (e.g., 320 °C) since the Δ*E*_fH_ decreases at higher temperature as discussed above. Our DFT results support the view that the topotactic phase transformation from the P to the H-BM phase can occur at a lower hydrogenation temperature (220 °C) compared to vacuum annealing (400 °C).^7^ It is also consistent with the absence of highly reduced phases at higher temperatures in the presence of hydrogen, as observed experimentally.

**Figure 4:**
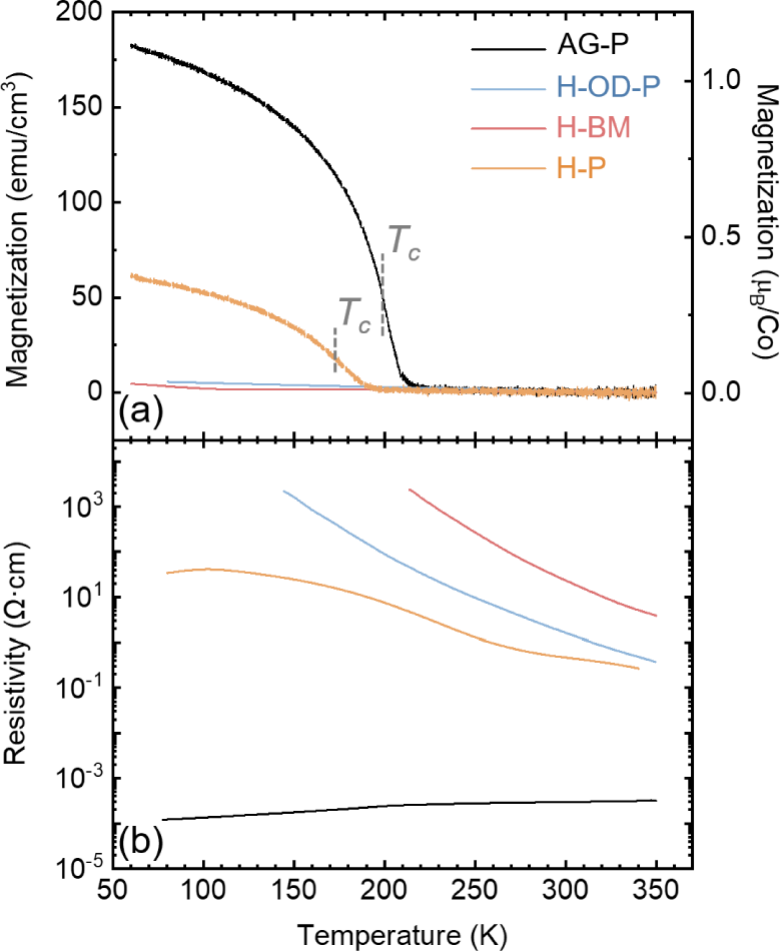
(a) Magnetization as a function of the temperature for AG-P and hydrogenated-LSCO thin films. The magnetization is normalized to the thin film volume (left *y*-axis) and number of Co ions (right *y*-axis). A magnetic field of 0.08 T was applied along the in-plane [100] substrate direction during the measurements. The vertical dashed gray lines mark *T*_c_ values. (b) Resistivity as a function of temperature.

Figure 4 presents the bulk magnetic and electrical characteristics of the AG-P and hydrogenated-LSCO thin films. The magnetization is normalized to the thin film volume (in emu/cm^3^) and the number of Co ions (in μ_B_/Co). All films were field-cooled in 0.08 T from 300 to 50 K to ensure the alignment of magnetic moments along the applied field direction. *T*_c_ is determined as the peak temperature of |d*M*/d*T*|. In the case of the AG-P film, a transition from FM to paramagnetic behavior, coupled with a metal-to-insulator transition, is observed around 200 K, which aligns well with the bulk LSCO value, as previously reported.^7,55^ Both H-OD-P and H-BM films display non-FM and insulating behaviors across the entire temperature range studied. The room temperature resistivity of the H-BM film (∼10^2^ Ω·cm) is approximately 6 orders of magnitude higher than that of the AG-P film, similar to the pure BM phase obtained from vacuum annealing.^7^ Meanwhile, below room temperature, the H-BM film exhibits higher resistivity than the pure BM phase, suggesting a larger band gap of the H-BM phase. This result is in agreement with findings of SrCoO_2.5_, with a smaller band gap than HSrCoO_2.5_ thin film with H^+^ ions bonded to O^2–^ ions.^2^ The H-P film displays reduced saturation magnetization, lower *T*_c_ (174 K), and an increase in resistivity (ρ_300K_ = 0.47 Ω·cm) compared to the AG-P film. The high resistivity and flattened *R(T)* in the low-temperature region of the H-P film suggest that charge carriers are localized due to inhomogeneities within the film, likely arising from the nonuniform distribution of oxygen vacancies and H^+^ ions. This result indicates that despite the H-P film retaining its perovskite crystal structure, the existence of even a small number of oxygen vacancies and H^+^ ions has a significant impact on its physical properties. The presence of these oxygen vacancies and the H^+^ ions hinders the double exchange interaction between Co^3+^ and Co^4+^ ions, resulting in diminished magnetization and increased resistivity. The suppression of double exchange has also been found in hydrogenated LSMO thin films with 3 orders of magnitude increase in resistivity due to lattice volume expansion that reduced overlap of Mn 3*d* and O 2*p* orbitals.^30^ The 4 orders of magnitude increase in room temperature resistivity between the AG-P and H-P film implies that this hydrogenation process can serve as a promising method for designing MIT devices without significant phase changes.

## Conclusions

In summary, LSCO thin films were hydrogenated in pure H_2_ gas from room temperature to 400 °C. The emergence of a H-BM phase with AFM/insulating properties was only achieved at intermediate temperatures from 170 to 290 °C compared to 400 °C or higher temperatures required under vacuum annealing conditions. This behavior was attributed to a lower oxygen vacancy formation energy in the presence of H_2_ molecules. XRD profiles, XA measurements, and DFT calculations collectively verified the formation of −OH bonds (insertion of H^+^ ions) and the loss of the O^2–^ ions in the vicinity of the −OH bond. An interesting observation was the preservation of the perovskite structure at higher hydrogenation temperatures (above 290 °C), distinct from the path toward more reduced phases, such as RP phases observed under vacuum annealing conditions. While the H-P phase maintains a close similarity with the crystal structure of the AG-P LSCO film, the magnetization was suppressed by ∼67% at 60 K and the room temperature resistivity increased by 4 orders of magnitude. This result implies the presence of a limited number of oxygen vacancies and the existence of H^+^ ions that impede double exchange interactions between Co ions. The versatility of the LSCO structure in adopting high-resistance phases, either through phase transformation to the BM phase or without substantial phase changes, underscores its potential for application in the fabrication of MIT devices tailored for instance for neuromorphic computing.

## Supporting Information Available

XRD curves of the LSCO thin films with hydrogenation temperatures up to 400 °C; HAADF-STEM images and EELS spectra of the O *K-* and Co *L*_2,3_-edges; DFT calculations of formation energy of hydrogen doping at different temperatures and oxygen vacancy formation energy with the presence of hydrogen (PDF)
