# Supplementary material for: Hydrogen-Induced Topotactic Phase Transformations of Cobaltite Thin Films
Source: J Phys Chem C Nanomater Interfaces. 2024 Sep 30;128(40):17124–33. doi: 10.1021/acs.jpcc.4c04098 (PMC11472462; doi:10.1021/acs.jpcc.4c04098)
Supplement: Supplementary file 1 — jp4c04098_si_001.pdf [file jp4c04098_si_001.pdf]

Supporting Information

Hydrogen-induced Topotactic Phase Transformations of Cobaltite Thin Films

*Mingzhen Feng,[1, ‡] Junjie Li,[2,3, ‡] Shenli Zhang,[4] Alexandre Pofelski,[5] Ralph El Hage,[2] Christoph Klewe,[6] Alpha T. N'Diaye,[6] Padraic Shafer,[6, †] Yimei Zhu,[5] Giulia Galli,[7] Ivan K. Schuller,[2,3] Yayoi Takamura[1, *]*

[1] Department of Materials Science and Engineering, University of California, Davis, Davis, California 95616, United States.
[2] Department of Physics, University of California, San Diego, La Jolla, CA 92093, United States.
[3] Materials Science and Engineering Program, University of California, San Diego, La Jolla, California 92093, United States
[4] Materials Science Division, Lawrence Livermore National Laboratory, Livermore, CA 94550, United States.
[5] Condensed Matter Physics and Materials Science Department, Brookhaven National Laboratory, Upton, New York 11973, United States.
[6] Advanced Light Source, Lawrence Berkeley National Laboratory, Berkeley, CA 94720, United States.
[7] Pritzker School of Molecular Engineering, University of Chicago, Chicago, IL 60637, United States.

Corresponding author email: ytakamura@ucdavis.edu

1. XRD profiles

The ω-2θ XRD curves shown in **Figure S1** provide further details on the temperature dependence of the hydrogenated topotactic transformation of the LSCO films. The half-order peaks originating from the H-BM phase (shaded in orange) only exist at intermediate hydrogenation temperatures (from 170 ºC to 290 ºC) and the perovskite peaks (shaded in blue) shift to lower angles (forming the H-OD-P phase) then nearly back to its initial 2θ value (H-P phase) with increasing temperature.

[‡]co-first author, [†]working at another affiliation, * corresponding author

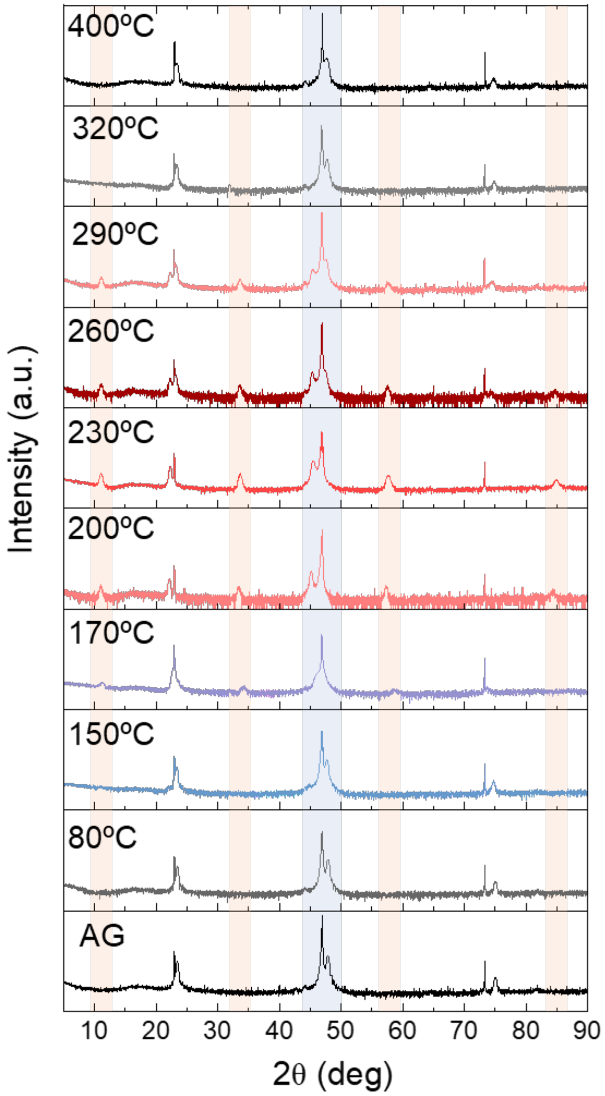

Figure S1. XRD profiles as a function of hydrogenation temperatures ranging from 80 ºC to 400ºC.

2. HAADF-STEM image

The top surface is P phase dominate while the bulk of the film is in the BM phase.

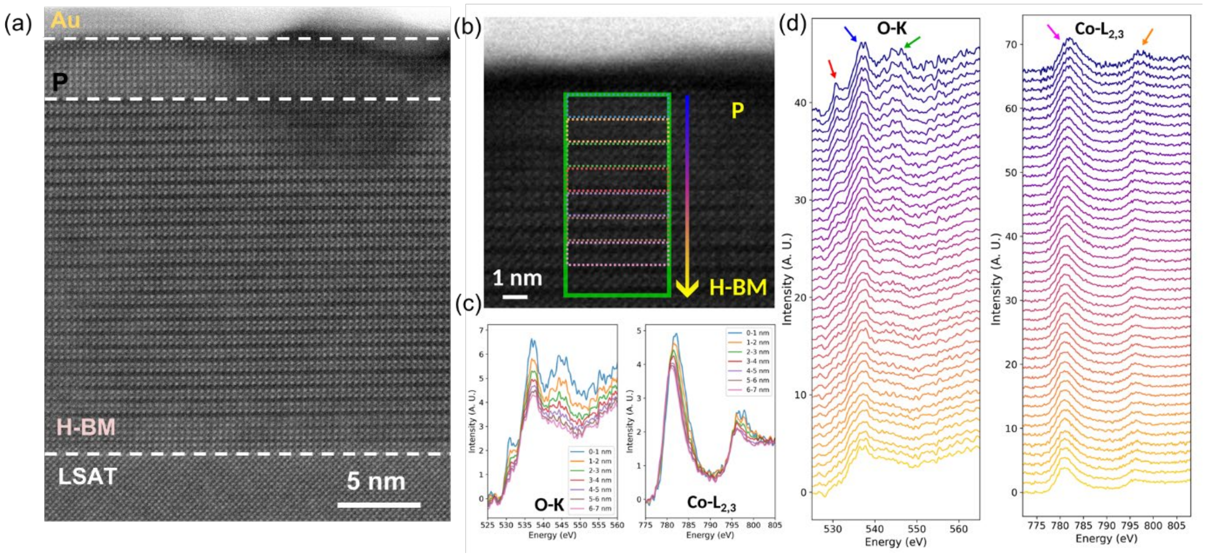

Figure S2. (a) HAADF-STEM image of the H-BM film highlighting the bulk H-BM and surface P phases, and the Au coating. (b) Cropped STEM ADF image focusing on the top part of the film. STEM EELS mapping performed in the green rectangle. (c) EELS spectra of the O-K and Co $L_{2,3}$ edges denoised using principal component analysis keeping the first 15 components. The EELS spectra are averaged over the areas shown in (b) following the same color code as in the plots. (d) Denoised EELS spectra averaged horizontally from the top to the bottom of the green box in (b). The colored arrows point to features of interests changing along the line profile.

3. DFT calculations

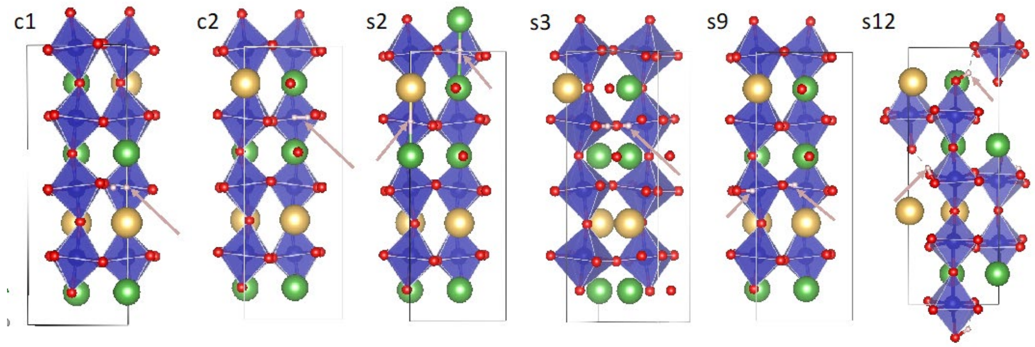

Figure S3. Representative sampled hydrogen doping configurations (initial atomic positions before relaxation). Color schemes for elements: La: green, Sr: yellow, Co: blue, O: red, H: white (pointed by arrows). c1 and c2 are H_2 molecular adsorption cases, where the H_2 molecules are in between 1:1 Sr:La ratio planes and 1:3 Sr:La ratio planes, respectively; H_2 molecules orient flat along the short axis. S2-s12: H_2 molecule decompose to two hydrogen atoms at different lattice sites: s2: two randomly placed hydrogen atoms in the unit cell; s3: two hydrogen atoms bond to oxygen ions that form a 90° in-plane O-Co-O bond angle; s9 and s10 (not shown): two hydrogen atoms bond to oxygen ions that form a 180° in-plane O-Co-O bond angle, with different Sr:La local ratio environment; s12: two hydrogen atoms bond to oxygen ions that form 180° out-of-plane O-Co-O bond angle. In-plane is formed by shorter axes and the out-of-plane direction is along the longer axis.

Table S1 Calculated formation energy of hydrogen doping (ΔE_{fH}) at different temperatures at 0.3 MPa (experimental pressure) after structural relaxation, using **Eqn. S1** and **S2**.

**Configurations**	ΔE_{fH} **(eV) 0 K**	ΔE_{fH} **(eV) 273 K**	ΔE_{fH} **(eV) 473 K**	**Co^{2+}: Co^{3+}: Co^{4+}**
c1 (relaxed to 90° HO-Co-OH bonding configuration)	**-1.33**	**-1.36**	**-1.38**	0:6:2
c2 (relaxed to 180° HO-Co-OH bonding configuration)	-0.99	-1.02	-1.04	0:6:2
s2	-0.52	-0.54	-0.56	0:5:3
s3	-1.11	-1.14	-1.16	1:3:4
s9	-1.02	-1.05	-1.07	0:6:2
s10	-1.21	-1.24	-1.26	0:5:3
s12 (relaxed to hydrogen bonded to oxygen ions at different octahedral layers)	-0.61	-0.64	-0.65	0:4:4

All the structures with hydrogen doping were initialized with ferromagnetic ordering, and relaxation of inner atomic coordinates were performed without unit cell volume optimization. The formation energy of hydrogen doping ΔE_{fH} is calculated as:

$$\Delta E_{fH} = E_{N+i} - E_N - i * \mu_H \quad \text{(Eqn. S1)}$$

$$\mu_{\mathrm{H}} = \frac{1}{2}\mu_{\mathrm{H}_2} = \frac{1}{2} * (E_{H_2}^{\mathrm{total}} + \mathrm{k}Tln\left(\frac{p}{p^0}\right)) \quad \text{(Eqn. S2)}$$

where E_{N+i} the total energy of relaxed structures after doping with i number of hydrogen atoms, E_N the total energy of the pristine structure, and μ_{H} the chemical potential of hydrogen atom. We calculated ΔE_{fH} at 0 K with $E_{H_2}^{\mathrm{total}}$ term (total energy of a hydrogen molecule), and then add temperature *T*, H_2 partial pressure *P* effect through $\mathrm{k}Tln\left(\frac{p}{p^0}\right)$, where p^0=1 atm.

The ratio between the number of Co^{2+}: Co^{3+}: Co^{4+} valence states are obtained from the calculated magnetic moment of Co ions, where we have assigned magnetic moment <1 μ_B to be Co^{2+} (spin state S=1), between 1~2 μ_B to be Co^{3+} (spin state S=2) and >=2 μ_B to be Co^{4+} (spin state S=3). Such an assignment gives the correct 5 Co^{3+}: 3 Co^{4+} ratio in the pristine structure where we have 5 La:3 Sr.

Table S2 Calculated oxygen vacancy formation energy (ΔE_{fO}) with the presence of hydrogen after cell optimization, using **Eqn. S3** and **S4**.

**Configurations**	ΔE_{fO} **(eV) 0 K**
no hydrogen presence	0.98
oxygen attached to H is removed	0.95
oxygen next to the OH bond is removed	-0.43
oxygen in the neighboring octahedral CoO_6 layers to the OH bond is removed	-0.24
oxygen in the next neighboring octahedral CoO_6 layers to the OH bond is removed	0.95

All the structures with hydrogen doping were initialized with ferromagnetic ordering, and the long axis of the cell is allowed to change during structural optimization, which corresponds to the varying out-of-plane axis in the thin-film samples. Similar to Eqn. S1 and S2, here the formation energy of oxygen vacancy formation energy ΔE_{fO} with the presence of hydrogen doping is calculated as:

$$\Delta E_{fO} = E_{N-j+i} - E_N + j * \mu_O - i * \mu_H \qquad \text{(Eqn. S3)}$$

$$\mu_{\mathrm{O}} = \frac{1}{2}\mu_{\mathrm{O}_2} = \frac{1}{2} * (E_{O_2}^{\mathrm{total}} + \mathrm{k}Tln\left(\frac{p}{p^0}\right)) \qquad \text{(Eqn. S4)}$$

where E_{N-j+i} the total energy of relaxed structures after removing i number of oxygen atoms while keeping j number of hydrogen atoms, E_N the total energy of the pristine structure, and μ_{O} the chemical potential of hydrogen atom. We calculated ΔE_{fO} at 0 K with $E_{H_2}^{\mathrm{total}}$ term (total energy of an oxygen molecule in triplet spin state).
